# Tailoring fungal morphology of *Aspergillus niger* MYA 135 by altering the hyphal morphology and the conidia adhesion capacity: biotechnological applications

**DOI:** 10.1186/2191-0855-3-27

**Published:** 2013-05-20

**Authors:** Verónica Leticia Colin, Mario Domingo Baigorí, Licia María Pera

**Affiliations:** 1 PROIMI-CONICET, Morphogenesis and Fermentation Lab, Av. Belgrano y Pasaje Caseros, San Miguel de Tucumán, Tucumán T4001 MVB, Argentina; 2 Facultad de Bioquímica, Química y Farmacia, Batalla de Ayacucho 471, San Miguel de Tucumán, Tucumán, T4000INI, Argentina

**Keywords:** *Aspergillus niger*, Culture conditions, β-N-Acetyl-D-Glucosaminidase, Hyphal morphology, Conidia adhesion, Pellets formation, Metabolite production

## Abstract

Current problems of filamentous fungi fermentations and their further successful developments as microbial cell factories are dependent on control fungal morphology. In this connection, this work explored new experimental procedures in order to quantitatively check the potential of some culture conditions to induce a determined fungal morphology by altering both hyphal morphology and conidia adhesion capacity. The capacity of environmental conditions to modify hyphal morphology was evaluated by examining the influence of some culture conditions on the cell wall lytic potential of *Aspergillus niger* MYA 135. The relative value of the cell wall lytic potential was determined by measuring a cell wall lytic enzyme activity such as the mycelium-bound β-N-acetyl-D-glucosaminidase (Mb-NAGase). On the other hand, the quantitative value of conidia adhesion was considered as an index of its aggregation capacity. Concerning microscopic morphology, a highly negative correlation between the hyphal growth unit length (l_HGU_) and the specific Mb-NAGase activity was found (r = -0.915, P < 0.001). In fact, the environment was able to induce highly branched mycelia only under those culture conditions compatible with specific Mb-NAGase values equal to or higher than 190 U g_dry_._wt_^-1^. Concerning macroscopic morphology, a low conidia adhesion capacity was followed by a dispersed mycelial growth. In fact, this study showed that conidia adhesion units per ml equal to or higher than 0.50 were necessary to afford pellets formation. In addition, it was also observed that once the pellet was formed the l_HGU_ had an important influence on its final diameter. Finally, the biotechnological significance of such results was discussed as well.

## Introduction

Filamentous fungi have been extensively used in biotechnological processes as cell factories due to the metabolic versatility of this group of microorganisms. They are known for their capacity to secrete high levels of enzymes, antibiotics, vitamins, polysaccharides and organic acids (Meyer 2008). Mycelia are also suitable for application in treatment of dye and textile wastewater (Romero et al. 2006; Blánquez et al. 2007). However, one particular difficulty with this kind of microorganisms centers on their morphological form. The observed hyphal morphology can vary from linear filaments to highly branched structures, while growth morphologies in submerged culture varying from compact pellets to dispersed mycelia. Both microscopic and macroscopic morphology affects the broth rheology and in this turn may affect product yield (Grimm et al. 2005). A number of culture conditions have been identified as having the potential to bring about substantial change in fungal morphology. It is well documented that morphology is influenced by medium composition, pH, temperature, additives (surfactants, chelators, polymers), inoculum and dissolved oxygen and carbon dioxide (Papagianni 2004). However, it has to be mentioned that much of the published work is descriptive, with relatively little attempt made to obtain quantitative work related to the control of fungal behavior in liquid culture. Thus, understanding the growth and morphological development during submerged cultures is therefore, an important part in studies of the fermentation processes of filamentous fungi (Pera et al. 2008, 2010).

Dispersed mycelial growth is preferred for many processes, but increases the viscosity of the medium and can wrap around impellers, cause blockages and spread into sampling and overflow lines (Prosser and Tough 1991). As a valuable strategy to overcome such obstacles, several authors have reported the use of immobilized mycelium (Papagianni and Mattey 2004; Mizunuma et al. 2007). Alternatively, growing the organism in the pelleted form also removes these problems and facilitates downstream processing by simplifying solid–liquid separation procedures. However, big pellets may often result in the diffusional limitation of oxygen and other nutrients. These limitations can finally induce autolytic processes within the inner part of those pellets. Therefore, the ability to obtain and control a certain pellet size would be a key to increase the mycelium productivity. As an interesting approach in this direction, a low hyphal growth unit length (l_HGU_) has been associated with the formation of clumps smaller than those formed at a high l_HGU_ (Bocking et al. 1999). Moreover, although the exact process of pellets formation is not fully understood, the initial step of some pelletization events is suggested to involve conidia aggregation (Prosser and Tough 1991). In this connection, quantitative values of conidia adhesiveness could be valuable information.

On the other hand, the cell wall is a complex and dynamic structure and site of diverse enzyme activities. The balance between wall synthesis and wall lysis is critical, since wall synthesis in the absence of lysis can cause excessive wall thickening and possible growth arrest, whilst lysis in the absence of synthesis would produce bursting of the cell. Thus, within permissible limits, the net balance between wall synthesis and wall lysis will exert a marked influence on cell growth and hyphal morphology (Grove 1978). In this connection, the activity β-N-Acetyl-D-glucosaminidase (EC 3.2.1.30) (NAGase) from *Aspergillus niger* MYA 135 was chosen as a relative marker of the wall lytic potential (Bartnicki-García and Lippman 1972). This enzyme was isolated, purified and characterized, and dose–response experiments of those effectors that influence hyphal morphology of *A. niger* MYA 135 were also conducted (Pera et al. 1997). In this work, the mycelium-bound NAGase (Mb-NAGase) activity was used as a biochemical marker of the wall lytic potential to quantitatively check the capacity of some culture conditions to induce a desired hyphal morphology. To this end, the influence of environmental conditions on both hyphal morphology and cell wall weakness was firstly evaluated. Later, a basic mineral medium at different temperatures as well as an aliquot of several culture media was exposed to a Mb-NAGase activity, and then the specific activity was calculated and related to the resulting hyphal morphology obtained under each assayed condition after 72 h of cultivation.

Thus, a quantitative assessment of hyphal morphology and conidia adhesion capacity of *A. niger* MYA 135 under different environmental conditions, and how those parameters affect the macroscopic morphology were the main goals of this study.

## Materials and methods

### Microorganisms and culture conditions

*Aspergillus niger* ATCC MYA 135, formerly *A. niger* 419 from our own culture collection, was used throughout this work. To produce conidia, the microorganism was grown on potato dextrose agar (PDA) for 10 to 20 days at 30°C. The PDA medium contained (g l^-1^): infusion from 300 g potatoes; dextrose, 10.0; agar-agar, 20.0. The culture flasks were incubated to a final concentration of about 10^5^ conidia ml^-1^. The basic culture medium (BM) contained (g l^-1^): sucrose, 10; KH_2_PO_4_, 1; NH_4_NO_3_, 2; MgSO_4_, 0.2; CuSO_4_, 0.06. All parameters measured from mycelial pellets grown in BM at 30°C and initial pH 5 were considered as a reference, because it was possible to detect significant changes of them above or below to these determined under the reference conditions. The cultivations were conducted in 50 or 500 ml conical flasks, containing 10 or 100 ml of BM, respectively, on an orbital shaker at 200 rpm during 72 h. The effect of modification in the environmental conditions on biomass production, fungal morphology and conidia adhesion was tested by changing either the temperature of incubation or the initial pH of the medium as well as by the addition of either 0.5 g l^-1^ CaCl_2_ or 1 g l^-1^ FeCl_3_ to the BM. At the end of the incubation period the biomass was determined by drying washed mycelia at 105°C to constant mass.

### Microscopic and macroscopic observations

Cell morphology was analyzed with Nomarski differential interference contrast (DIC) optics, utilizing a Nikon ECLIPSE 80i microscope with a 100× 1.3 NA objective.

Morphological parameters such as pellet diameter, hyphal growth unit length (l_HGU_) and hyphal diameter as well as the numbers of pellets per liter were determined after 72 h of incubation by using the Nikon EclipseNet software package version 1.20.

For measurement hyphal lengths fragments, mycelia were collected, immediately suspended in distilled water and disintegrated with a dissection needle, until microscopic examination showed that the fungal hyphae were separated from each other. The l_HGU_ was calculated by dividing the total length for each organism by the number of tips. For each sample, an appropriate number of mycelial elements was analyzed (n ≥ 20), and the mean values calculated. Lengths were obtained by skeletonizing hyphal trees and using a pre-set calibration (in μm). Samples were analyzed at a total magnification of 400 ×. In addition, the arithmetic values of all morphological parameters passed the Anderson-Darling normality test.

The final macroscopic morphology (pellets or dispersed mycelium) obtained under each culture condition tested was also evaluated.

### Production of a wall lytic potential biomarker

Mycelial pellets produced in BM at initial pH 5, during 72 h, at 30°C were directly used as a source of Mb-NAGase activity.

### Determination of Mb-NAGase activity as a wall lytic potential biomarker

The response of the wall lytic potential biomarker (Mb-NAGase activity) towards different *in vitro* environmental conditions was evaluated in a reaction system as follows: to 500 μl of each culture medium without inoculum, about 0.010 g of wet mycelium (Mb-NAGase activity), and 50 μl of 3.6 mM p-nitrophenyl-N-acetyl-β-D-glucosaminide were added. The mixture was shaken at 200 rpm with magnetic bars at the corresponding temperature and, after addition of 1000 μl of 0.25 M Na_2_CO_3_ to stop the reaction, was centrifuged at 5000 g for 10 min. The optical density at 405 nm was measured in the supernatant, and enzyme activity was then calculated and related to biomass dry weight. A calibration curve was generated with wet and dry mycelia (R^2^ = 0.973).

Note that the aliquot of each medium was taken before inoculation. This is because conidia also have an ungerminated conidia-bound NAGase activity (data not shown).

One unit of enzyme activity was defined as the amount of biocatalyst that released 1 μmol of *p*-nitrophenol per minute.

### Determination of conidia adhesion

The adhesion capacity of *A. niger* conidia was assayed using 96-wells polystyrene microtiter plates as a substrate. According to the experimental protocols, 6 ml of culture samples were periodically withdrawn at 0, 2, 4 and 6 h of cultivation, centrifuged 10 min at 12,000 rpm, resuspended in 600 μl by using their own medium and dispensed in three wells. Plates were incubated at the same temperature at which the fungus was developed on an orbital shaker at 200 rpm, and then the medium containing most of the unbound conidia was removed by inverting the plate onto an adsorbent pad. Conidia adhesion was quantified with the protein stain sulforhodamine B (SRB). Conidia were fixed by incubation at 4°C for 1 h after the addition of 200 μl of 10% (v/v) trichloroacetic acid. The trichloroacetic acid was removed, the wells were washed five times with water, and the plates were allowed to dry in a chemical hood. Conidia were stained for 30 min at room temperature by the addition of 200 μl of a 0.4% (w/v) solution of SRB in 175 mM acetic acid to each well. The stain was removed by aspiration, and the plates were washed four times with 175 mM acetic acid. Then, the plates were dried by using a microwave oven for 15 min. The bound dye was extracted by adding 200 μl of 10 mM unbuffered Tris base (pH 10.1) to each well and shaking the plates for 10 min at 250 rpm. The resulting pink color in the wells was quantified by measuring the absorbance at 570 nm (Slawecki et al. 2002). The adhesion unit (U) was defined as the amount of adherent substance that produces an increase in absorbance at 570 nm of 0.01 per min. The conidia adhesion was expressed as U per ml of culture. For each environmental condition tested, a minimum of nine replications were made.

### Influence of conidia-aging on adhesion

The influence of conidia-aging on adhesion to polystryrene wells was firstly tested after 3 h of incubation at 30°C by using ungerminated conidia from 2 to 40-day-old suspended in BM at initial pH 5. Secondly, adhesion units per ml of either 10 or 30-day-old conidia were also determined during the first hours of cultivation (0, 2, 4 and 6 h).

### Influence of azide on conidia adhesion

Conidia were suspended in BM, in BM supplemented with 2 mM azide, in water as well as in water supplemented with 2 mM azide, and they were incubated at 30°C. Culture samples were periodically withdrawn and the adhesion units per ml detected during the first hours of cultivation were determined as described above.

### Statistical analysis

Statistical analyses were performed using the Infostat (version 2004; Grupo InfoSat, FCA, Universidad Nacional de Córdoba, Córdoba, Argentina) and the Minitab (version 14; Minitab Inc) softwares for windows. Statistical significance values of the means were evaluated using a one-way analysis of variance. Subsequent comparisons were performed using Tukey’s post-hoc test. Results were presented as the mean ± SD. Differences were accepted as significant when P < 0.05. In addition, the Durbin-Watson test was used to detect the presence of autocorrelation in the residuals from a regression analysis.

## Results

The huge efforts in the characterization of fungal morphology show an evident connection between the operating environment of the bioprocess and the morphology and metabolic properties of individual cells (Krull et al. 2013). In this work, new experimental procedures were explored in order to quantitatively check the potential of some culture conditions to induce a determined fungal morphology by altering both hyphal morphology and conidia adhesion capacity.

### Influence of environmental conditions on biomass production and fungal morphology

**Figure 1:**
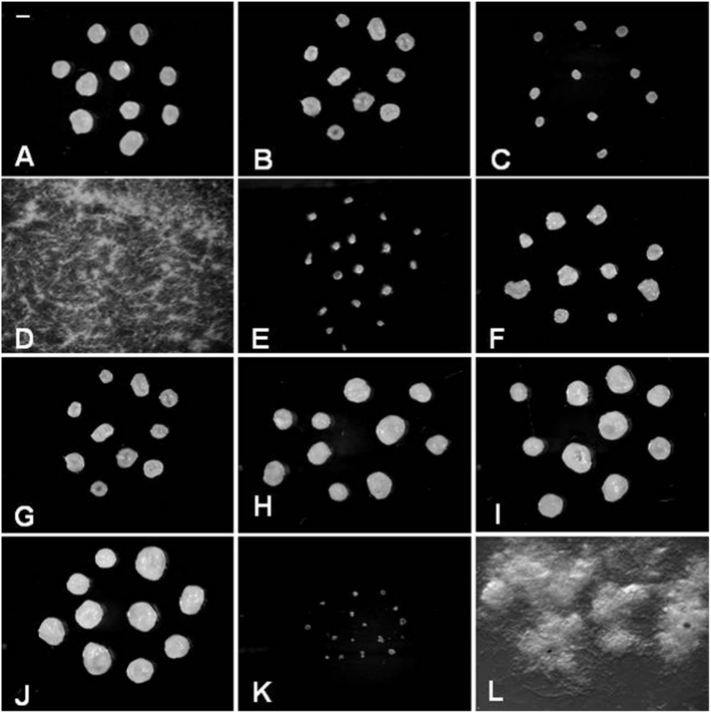
**Macroscopic morphology of *A. niger *MYA 135 in submerged fermentation. **Pictures show fungal mycelia developed in basic medium at 25°C (**A**), 30°C (**B**), 37°C (**C**), pH 2(**D**), pH3 (**E**), pH4 (**F**), pH5 (**G**), pH6 (**H**), pH7 (**I**) and pH8 (**J**) as well as those obtained in the presence of either 0.5 g l^-1 ^CaCl_2 _(**K**) or 1.0 g l^-1^ FeCl_3 _(**L**). Bar represents 1 mm. These images were taken after 72 h of cultivation.

The influence of several environmental conditions such as agitation, pH, temperature, ions, etc., on fungal morphology was examined by dose–response experiments (data not shown). As expected, microscopic and macroscopic morphologies range from linear filaments to highly branched structures and from discrete pellets to dispersed mycelium (Figure 1), respectively. The most representative morphological patterns were chosen to conduct this work.

**Figure 2:**
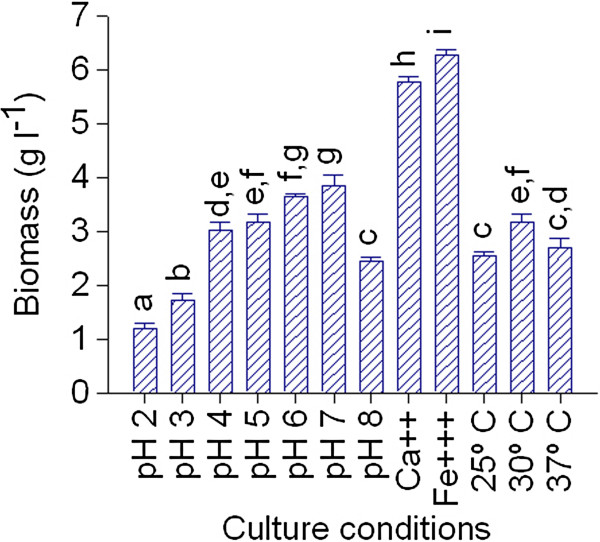
**Biomass production during a submerged process of *A. niger *MYA 135 under different culture conditions. **Error bars represent the standard deviation calculated from at least three independent experiments. Bars with the same letter are not significantly different. These data were obtained after 72 h of cultivation.

**Figure 3:**
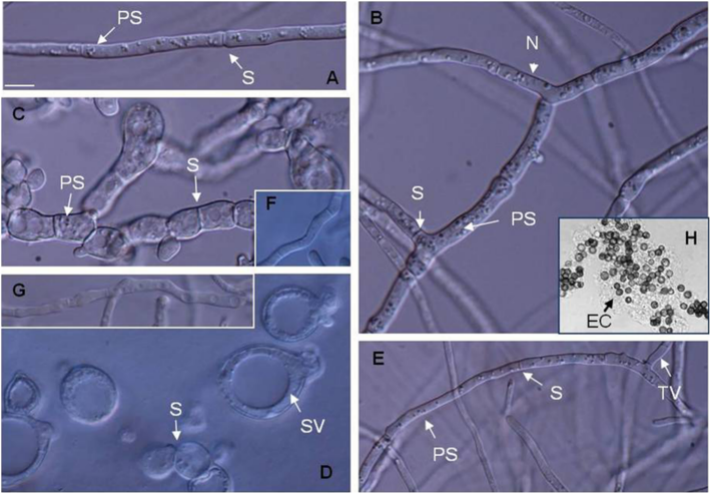
**Microscopic morphology of *A. niger *MYA 135 in submerged fermentation. **Differential interference contrast images of cells developed in a basic medium at 25 (**A**), 30 (**B**) and 37°C (**C**) as well as those obtained in the presence of either 0.5 g |^-1^ CaCl_2 _(**D**) or 1 g |^-1 ^FeCl_3 _(**E**). Restored cells observed at 37°C (**F**) or in the presence of 0.5 g |^-1 ^CaCl_2 _(**G**) with the supplementation of 1.5 M NaCl. These images were taken after 72 h of cultivation. Extracellular compound (EC) produced during the early stages of pellets formation (**H**). All Panels are at the same magnification as **A**. Bar represents 10 μm. SV (Spherical Vacuole), TV (Tubular Vacuole), S (Septum), N (Nucleus), PS (Punctuate Structures).

Firstly, as culture factors defining the physiology and the morphology of fungal cells, an initial analysis of *A. niger* MYA 135 grown in submerged culture was performed in twelve different defined culture conditions. After 72 h of incubation in BM (basic mineral medium), an optimal growth in the pH range of 6.0 – 7.0 was observed (Figure 2). The hyphal polarity was not altered within the pH range from 2 to 8 (data not shown). In contrast, the temperature of incubation heavily modified the cell morphology. At 25°C, DIC images revealed long and straight hyphal compartment with regular distributed clusters of punctate structures (Figure 3A). These structures, which may be small vacuoles (Shoji et al. 2006) as well as the frequencies of hyphae ramification were readily increased at 30°C (Figure 3B). Finally, cells developed at 37°C were swollen and abnormally shaped showing some bifurcations in the apex and septa closely placed (Figure 3C).

Compared to the mycelial yield obtained under reference culture conditions (BM at 30°C and initial pH 5), in BM supplemented with 0.5 g l^-1^ CaCl_2_ or 1 g l^-1^ FeCl_3_ the biomass production was increased by 45 and 49%, respectively (Figure 2). However, their cellular morphologies were completely different. In the presence of CaCl_2_ numerous bulbous cells containing a large spherical vacuole and intercalary compartments abnormally short, appearing “barrel-shaped” in some cases, were observed (Figure 3D). Whereas, cells obtained in the presence of FeCl_3_ exhibited septa at a sufficient distance from one another, tubular vacuoles and, punctuated structures similar to those observed in hyphae grown at 25°C (Figure 3E).

Bulbous cells obtained in the presence of 0.5 g l^-1^ of CaCl_2_ appear to be bigger than those developed at 37°C. This fact was indicated by their 95% confidence intervals for the mean main diameter, that range from 15.24 and 12.59 μm to 17.81 and 14.44 μm, respectively, as well as by the relative position of the peaks for the fitted normal distribution (data not shown). In addition, both phenotypes were restored in a high osmolarity medium, suggesting the presence of a weakened cell wall (Figure 3F and 3G). No bulbous cells were observed in a medium supplemented with 1.5 M NaCl.

It is also important to mention that the morphological characteristics of growth were already apparent after about 24 h of incubation (data not shown).

On the other hand, during the early stages of the pelleted form of growth, conidia from *A. niger* MYA 135 produced a presumptively adhesive extracellular compound, probably an exopolysaccharide, visible under the optical microscope (Figure 3H). This amorphous material was not observed when the microorganism grew mainly as dispersed mycelia. Eventually, conidia could form small conglomerates, which grew laxly, along with hyphae originated from single, i.e., non associated conidia, apparently free of extracellular compound (data not shown).

**Figure 4:**
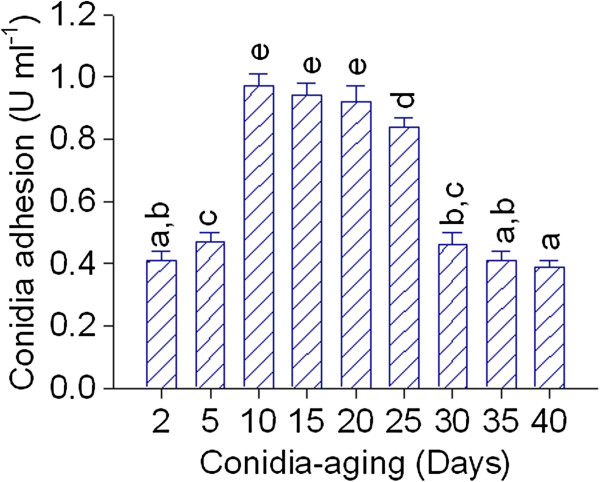
**Effect of *A. niger *MYA 135 conidia-aging on adhesion to polystyrene well. **Error bars represent the standard deviation calculated from at least three independent experiments. Bars with the same letter are not significantly different.

In addition, as conidia-aging can affect their adhesion capacity (Mercure et al. 1994), the initial adhesiveness of ungerminated conidia from 2 to 40 day-old was firstly evaluated by their affinity to polystyrene wells. As shown in Figure 4, ungerminated conidia from 10 to 20 day-old showed a maximum adherence level. Thus, the conidia adhesive competence decreases in conidia younger than 10 day-old as well as in conidia older than 20 day-old. Besides, to determine the effect of the conidia aging on fungal growth, BM was inoculated with either 10 or 30 day-old conidia. Samples were periodically withdrawn and then adhesion units per ml were determined. Although the adhesion pattern was almost the same in both cases, old conidia always displayed a lower adhesion capacity to polystyrene wells (data not shown). Interestingly, pellet diameters measured after 72 h of cultivation were also significantly different (P < 0.001). Pellets developed from 10 day-old conidia were 36% bigger than those developed from 30 day-old conidia showing diameters of 1.58 ± 0.20 and 1.01 ± 0.13 mm, respectively. This is an important result because the final pellet diameter also depended on the inoculum age. Therefore, all subsequent assays were conducted with conidia from 10 to 20 day-old.

Moreover, as the capacity of some fungi conidia to adhere is affected by their exposure to respiration inhibitors (Mercure et al. 1994), the influence of azide on adhesion was also examined. Thus, under our assay conditions, the presence of 2 mM azide decreased the initial conidia adhesion by 38%. This adhesion capacity remained unchanged after 6 h of cultivation. Interestingly, over the same period of time both conidia suspended in distilled water (or drinking water) and conidia suspended in distilled water supplemented with 2 mM azide did not adhere to polystyrene at all (Table 1).

**Table 1:** **Effect of sodium azide on *A. niger *MYA 135 conidia adhesion**

**Culture conditions**	**Time course of adhesion capacity (U ml**^**-1**^**) ± SD**			
	**0 h**	**2 h**	**4 h**	**6 h**
MB (basic medium)	0.89 ± 0.03	1.84 ± 0.04	2.10 ± 0.08	2.52 ± 0.16
MB + 2 mM NaN_3_	0.55 ± 0.03	0.54 ± 0.02	0.54 ± 0.02	0.57 ± 0.05
H_2_O_d_	0	0	0	0
H_2_O_d_ + 2 mM NaN_3_	0	0	0	0

Finally, as expected, the final pellet diameter as well as the number of pellets per liter was also affected by the environmental conditions (Table 2).

**Table 2:** **Effect of environmental conditions on pellet diameter and pellets number in cultures of *A. niger *MYA 135**

**Culture conditions**	**Pellet diameter (mm)**	**Pellets l**^**-1 **^**(x10**^**3**^**)**
pH_i _2	DM	DM
pH_i _3	0.28 ± 0.06 b	275 ± 3 f
pH_i _4	1.12 ± 0.11 d	60 ± 3 d
pH_i _5	1.31 ± 0.30 e	55 ± 3 c, d
pH_i _6	1.44 ± 0.32 f	45 ± 3 b
pH_i _7	2.51 ± 0.36 g	22 ± 2 a
pH_i_ 8	2.75 ± 0.32 h	19 ± 2 a
25ºC	1.35 ± 0.11 e, f	41 ± 4 b, c
30ºC	1.31 ± 0.30 e	55 ± 3 c, d
37ºC	0.46 ± 0.09 c	249 ± 6 e
CaCl_2 _(0.5 g |^-1^)	0.14 ± 0.04 a	328 ± 8 g
FeCl_3 _(1.0 g |^-1^)	DM	DM

Error represents the standard deviation calculated from at least three independent experiments. Data with the same letter are not significantly different. The results were obtained after 72 h of cultivation. DM (Dispersed Mycelium), pH_i_ (initial pH).

### Influence of environmental conditions on a mycelium-bound β-N-acetyl-D-glucosaminidase activity and its relationship with hyphal morphology

The cell wall is essential for maintaining the osmotic balance and the shape of the cell. So, considering that a weakness in the cell wall was observed under determined culture conditions (Figure 3F and 3G) and that the site of new branch must be weakened to permit the formation of a new apex, the response of a wall lytic enzyme such as the mycelium-bound β-N-Acetyl-D-glucosaminidase (Mb-NAGase) activity was evaluated as a relative marker of the wall lytic potential.

**Figure 5:**
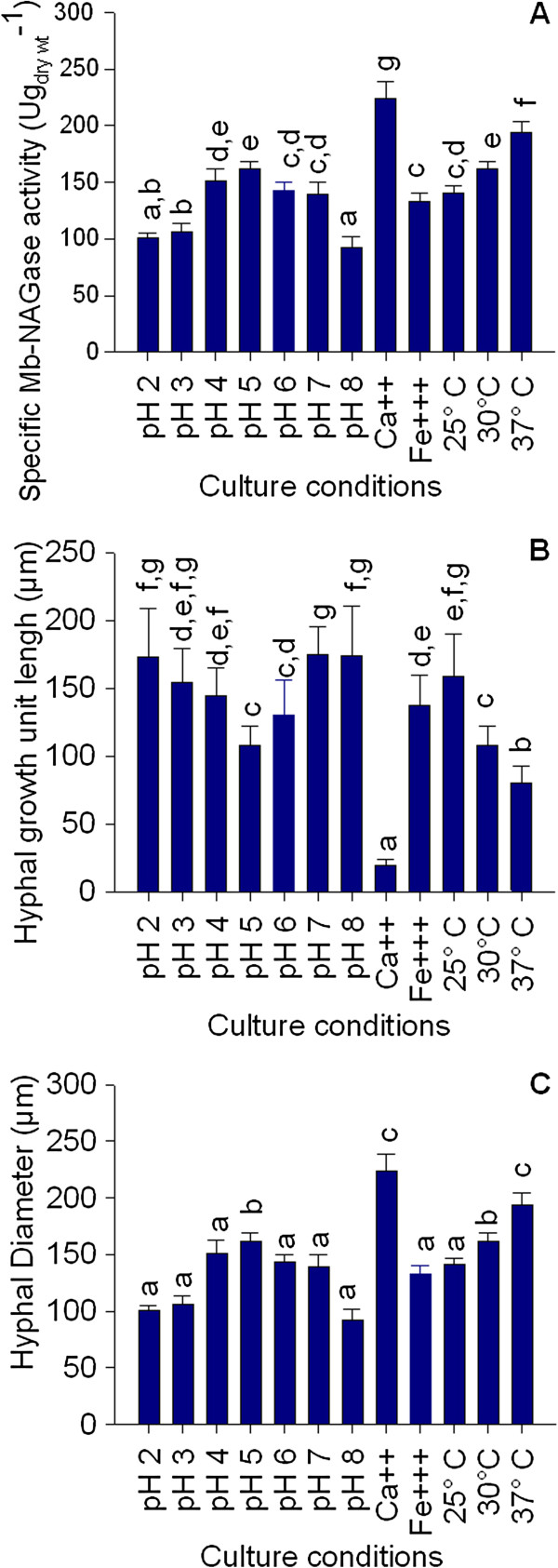
**Effect of culture conditions on specific Mb-NAGase activity (A), hyphal growth unit length (B) and hyphal diameter (C) from *A. niger *MYA 135. **Error bars represent the standard deviation calculated from at least three independent experiments. Bars with the same letter are not significantly different.

The specific Mb-NAGase activity was found to be most active in reaction mixtures containing BM in the pH range of 4.0 – 5.0. As expected, within the range of temperature tested, an increase of incubation temperature also causes an increase of enzyme activity. Finally, the presence of metal ions also influenced the specific Mb-NAGase activity. Reaction mixture contained BM supplemented with 0.5 g l^-1^ of CaCl_2_ significantly enhanced this activity. However, in the presence of 1 g l^-1^ FeCl_3_ an opposite catalytic response was detected (Figure 5A). On the other hand, it was also possible to establish that the specific Mb-NAGase activity determined in the presence of 0.5 g l^-1^ CaCl_2_ was significantly higher than that observed at 37°C. Interestingly, this biomarker response appears to be directly related to the size status of bulbous cells obtained under those environmental conditions. These data show a specific Mb-NAGase activity which can be modified by the initial culture conditions.

In order to quantitatively examine how some environmental conditions affect the microscopic morphology, the l_HGU_ and the average hyphal diameter of the mycelium obtained in each batch after 72 h of cultivation were determined. In this analysis, the bulbous cell diameters were not included. The l_HGU_ and the average hyphal diameter for the *A. niger* MYA 135 growing under reference conditions (BM at 30°C and initial pH 5) were 108 ± 14 μm and 3.5 ± 0.2 μm, respectively. These values were significantly modified by the pH of the medium, the presence of metal ions as well as by the temperature of incubation (Figure 5B and 5C).

**Figure 6:**
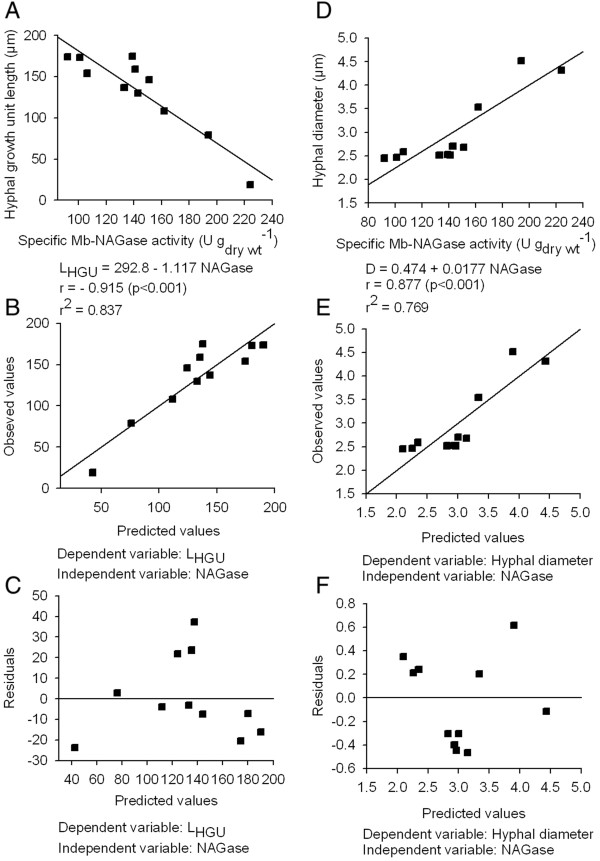
**Relationships between the specific Mb-NAGase activity and the morphological indices in *A. niger *MYA 135. **Correlation of specific Mb-NAGase activity with |_HGU _(**A**) and with the hyphal diameter (**D**). Observed versus predicted values of correlation between |_HGU _(**B**) or hyphal diameter (**E**), and specific Mb-NAGase. Residuals versus predicted values of correlation between |_HGU_ (**C**) or hyphal diameter (**F**), and specific Mb-NAGase. Mb-NAGase (Mycelium-bound β-N-Acetyl-D-glucosaminidase), |_HGU _(hyphal growth unit length), D (Hyphal diameter).

As it was mentioned before, the initial culture pH seems to have no influence on hyphal polarity. However, the l_HGU_ of fungal mycelium significantly fell with increasing the pH of the medium from 4 to 5 (Figure 5B). Above pH 5 a gradual increase of this morphological parameter was again detected. Interestingly, the average hyphal diameter was indistinguishable in mycelia developed at pH values below or above 5 (Figure 5C). Concerning the influence of metal ions on hyphal morphology, the l_HGU_ for the mycelium developed in the presence of CaCl_2_ was 86% lower than that developed in the presence of FeCl_3_, indicating that under the first culture condition the microorganism grew more densely branched. In addition, the hyphal diameter of cells grown in BM supplemented with CaCl_2_ was significantly higher than those grown in BM supplemented with FeCl_3_. Finally, the l_HGU_ decreased and the hyphal diameter increased both linearly, with increasing the temperature of incubation. To evaluate the relationship between these morphological parameters and the specific Mb-NAGase activity correlation studies were performed (Figure 6). Both l_HGU_ (r = -0.915; P < 0.001) (Figure 6A) and hyphal diameter (r = 0.877; P < 0.001) (Figure 6D) were highly correlated with the specific Mb-NAGase activity. Figure 6 also showed the plots of observed versus predicted values and residuals versus predicted values for the relationships between l_HGU_ and NAGase (Figure 6B and 6C) and hyphal diameter and NAGase (Figure 6E and 6F). In figures 6B and 6E, it was found that the points were aligned when compared with the adjusted straight line. In addition, the figures 6C and 6F allowed the verification of the random distribution of residues around zero, which is a requirement to obtain adequate models. Thus, under the tested assay conditions, all initial culture conditions that increased this NAGase source activity were able to decrease the l_HGU_ and to increase the hyphal diameter (Figure 5B and 5C). However, the environment was able to induce highly branched mycelia only under those culture conditions compatible with a specific Mb-NAGase value equal to or higher than 190 U g_dry_._wt_^-1^.

### Influence of environmental conditions on conidia adhesion and its relationship with pellets formation

Because conidia of *A. niger* MYA 135 are of the coagulative type, quantitative values of conidia adhesiveness and its relationship with pellets formation could be a valuable information.

**Figure 7:**
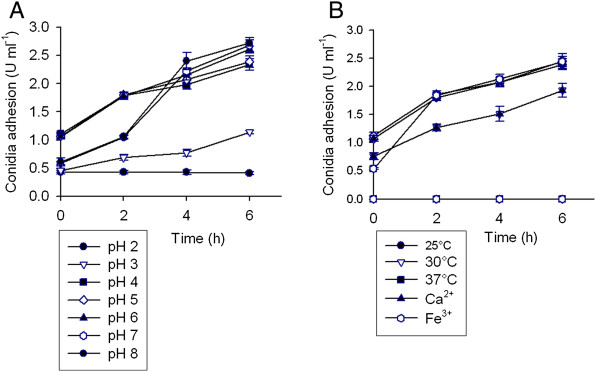
**Time course of *A. niger *MYA 135 conidia adhesion observed in basic medium (BM) at different initial pH (A), temperature of incubation and in the presence of either 0.5 g l**^**-1 **^**CaCl**_**2 **_**or 1 g l**^**-1 **^**FeCl**_**3 **_**(B). **Error bars represent the standard deviation calculated from at least three independent experiments.

As shown in Figure 7A, the initial pH of the BM strongly modified the adhesiveness of conidia. At initial pH 2 the conidia adhesion capacity determined at time 0 (0.43 ± 0.03 U ml^-1^) was not significantly changed during all the experiment long. In contrast, at higher pH values the adhesion units per ml increased over the time displaying different adherence patterns. Although at pH 3 the conidia adhesion looks delayed an increase of 148% was detected after 6 h of cultivation. The maximum level of initial conidia adhesion was observed at initial pH 4, 5 and 6. As expected, these values were increased in a time-dependent manner. However, after 6 h of cultivation adhesion units per ml reached by conidia incubated at pH 6 (2.61 ± 0.05 U ml^-1^) were significantly higher than those developed at initial pH 4 (2.34 ± 0.10 U ml^-1^) and 5 (2.39 ± 0.10 U ml^-1^). The adhesion pattern displayed by conidia incubated in BM either at initial pH 7 or 8 was completely different. The initial conidia adhesion decreased as the initial pH increased from 6 to 7 or 8. However, after 4 h of cultivation conidia adhesion in BM at pH 7 and 8 increased abruptly by 276 and 293%, respectively. In addition, pellet number per liter decreased following an increase of the culture pH value (Table 2).

The influence of Ca^++^ on the extent of conidia adhesion was interesting. Although in the presence of 0.5 g l^-1^ CaCl_2_ the conidia adhesion follows the same pattern to that observed under reference conditions (BM at 30°C and initial pH 5), the adhesion capacity was reduced by about 26% (Figure 7B). It was also observed a significantly reduction in mean pellet diameter and an increase in the average number of pellets per liter (Table 2).

On the other hand, at initial pH 2 or in BM supplemented with 1 g l^-1^ FeCl_3_ dispersed form of growth were observed (Figure 1D and 1L).

Finally, the temperature of incubation did not appear to significantly affect conidia adhesion after 2 h of cultivation (Figure 7B). However, at 37°C, a significant reduction of pellet diameter as well as a significant increase of the number of pellets per liter was observed (Table 2). As it will be discussed below, these effects could be explained by the increase of the mycelia branching degree (Figure 5B).

It is well established that pellet diameter has an important influence in some biotechnological process. In fact, studies performed on *Aspergillus niger* NRRL-3 showed that the outer layer of the pellets is the productive fraction of the mycelium (El-Enshasy et al. 2006). In this work, statistical analyses were conducted to explore the relationships between pellet diameters measured after 72 h of cultivation and conidia adhesion capacity. As conidia display different adhesion patterns, no correlation between the conidia adhesion units per ml determined at time zero with final pellet diameter was found (r = 0.046; P = 0.900). However, a positive association was seen between final average pellet diameter and conidia adhesion units per ml determined after either 4 (r = 0.706; P = 0.023) or 6 (r = 0.721; P = 0.019) hours of cultivation. On the other hand, a simple linear regression considering conidia adhesion units per ml measured after 6 h of cultivation as the explanatory variable and final pellet diameter as the response variable produced a regression line that explains 52.0% of the variability of the data (R^2^ = 0.52; R^2^_(adj)_ = 0.46; P = 0.019) (Table 3).

**Table 3:** **Simple linear regression between pellet diameters *versus *conidia adhesion using *A. niger *MYA 135**

**Predictor**	**Coef**	**SE Coef**	**T**	**P**
Constant	-1.813	1.066	-1.70	0.127
Conidia adhesion^a^	1.3337	0.4532	2.94	0.019
Other parameters	S = 0.634256	R^2^ = 52.0%	R^2^_(adj)_ = 46.0%	DW = 1.59708

^a^ Conidia adhesion capacity determined after 6 h of cultivation.

Moreover, as it was mentioned before, the environmental conditions also affect the microscopic morphology (l_HGU_) of *A. niger* MYA 135 (Figure 5B). So, considering that the conidia adhesion units per ml measured after 6 h of cultivation and l_HGU_ were not highly correlated (r = 0.211, P = 0.558), the addition of the l_HGU_ variable to the regression equation was examined. Multiple linear regressions with pellet diameters versus conidia adhesion (6 h) and l_HGU_ explains now 82.6% of the variability of the data (R^2^ = 0.826; R^2^_(adj)_ = 0.777; P = 0.002) (Table 4). This analysis showed that both conidia adhesion capacity and hyphal morphology were among the governing factors affecting the final pellet diameter.

**Table 4:** **Multiple linear regression between pellet diameters *versus *conidia adhesion and hyphal growth unit length using *A. niger *MYA 135**

**Predictor**	**Coef**	**SE Coef**	**T**	**P**
Constant	-2.5595	0.7175	-3.57	0.009
Conidia adhesion^a^	1.1125	0.2982	3.73	0.007
l_HGU_^b^	0.010047	0.002860	3.51	0.010
Other parameters	S = 0.407917	R^2^ = 82.6%	R^2^_(adj)_ = 77.7%	DW = 1.87488

^a ^Conidia adhesion capacity determined after 6 h of cultivation.

^b ^l_HGU _measured after 72 h of cultivation.

## Discussion

Fungal morphology is often a bottleneck of productivity in several industrial processes; and in many cases, the problems generated by fungal development are solved based on operator experience. Thus, its control is a challenge not only in basic mycology but also in fungal biotechnology. Many studies have investigated the cellular and molecular components involved in shaping of fungal cell, but the mechanisms that govern and regulate polarized growth are still only partially understood (Rittenour et al. 2009; Amicucci et al. 2011). Concerning fungal development, it has been stated that for the production of some industrial metabolites specific growth morphology is necessary. In some processes, dispersed mycelium is required as in the production of penicillin from *Penicillium chrysogenum* and in the synthesis of pectinases by *Aspergillus niger*. While, pelleted growth is preferred for the production of citric and itaconic acids using *Aspergillus niger* and *Aspergillus terreous*, respectively (Žnidaršič and Pavko 2001; Papagianni 2004). Thus, since there is an abundance of literature on the optimal fungal morphology for a given bioprocess (Wucherpfennig et al. 2011), it was an important objective of this work to link fungal growth to quantitative determinations of both hyphal morphology and conidia adhesion capacity.

Concerning macroscopic morphology, the production of an adhesive compound could be one of the reasons for the initial aggregation of conidia to form pellet nuclei, enabling the further formation of pellets by the subsequent aggregation of small clamps of germinated conidia (Prosser and Tough 1991). In this connection, although several lines of evidence suggest that an extracellular compound secretion is involved in the conidia aggregation of many fungal species (Zelinger et al. 2006; Fontaine et al. 2010), Deyesen and Nielsen (2003) also explain this process as a result of hydrophobic and electrostatic interactions by using *Aspergillus nidulans* as a model. Interestingly, when *A. niger* MYA 135 grew mainly as dispersed mycelia the presence of an extracellular compound, if any, was only detected with electron microscope (data not shown). In addition, the production of an emulsifier activity was significantly increased (p < 0.05) under environmental conditions that support dispersed mycelial growth (Colin et al. 2010a). Similarly, Prosser and Tough (1991) reported that the addition of nonionic surfactants, e.g., Span, to shake-flask cultures of *A. niger* also decreases spore aggregation thus allowing for dispersed growth. These observations encourage us to quantify the conidia adhesion capacity under different environmental conditions.

Firstly, it is important to note that the age of conidia significantly affected the adhesive competence of ungerminated conidia from *A. niger* ( MYA 135. Like us, Amiri et al*.*^2005^) reported on a lower adhesion capacity to polystyrene of *Penicillium expansum* 40 day-old conidia, compared to the 15 day-old conidia. According to Smith et al. (1998), these results could be compatible with a decrease of the surface hydrophobicity of conidia ageing. However, these authors did not indicate what happens with conidia younger than 10 day-old.

Additionally, the capacity of ungerminated conidia from *A. niger* MYA 135 to adhere was affected by their exposure to a respiration inhibitor such as azide. This result suggested that the metabolism has a major role in the adhesion capacity of these conidia. However, it should be pointed out that conidia from *A. niger* MYA 135 could also have a preformed adhesive material (Schumacher et al. 2008) that is functional in BM at initial pH 5 and not in water. In contrast, the conidia adhesiveness of other filamentous fungi such as *Stagonospora nodorum* (Newey et al. 2007) and *Botrytis cinerea* (Doss et al. 1993) is not affected by this respiration inhibitor, showing that in these cases the metabolism is not required.

Interestingly, during the early stages of *A. niger* MYA 135 growth four differential responses concerning the conidia adhesion capacity over the time under different environmental conditions were observed such as no capacity to adhere, an initial adhesion capacity that is not significantly modified over the time, a conidia adhesion capacity that is gradually increased over the time and a conidia adhesiveness that is drastically increased over the time. In addition, this study also showed that conidia adhesion units per ml equal to or higher than 0.50 were necessary to afford pellets formation.

The pH value of the culture medium is a very important factor for several fungi to form pellets. In agreement with reports on *A. niger* growth morphology (Papagianni 2004), *A. niger* MYA 135 pellets formation decreased with increasing initial acidification of the culture medium. Besides, as it was shown in this work, the conidia adhesion capacity appears to be pH dependent as well.

Fungal morphology has been established as one of the key bioprocess parameters. In this context, the presence of metal ions in submerged fermentations significantly changes the macroscopic morphology and the metabolite production of *A. niger* MYA 135. Under citric acid culture conditions, the presence of 0.5 g l^-1^ CaCl_2_ in the fermentation medium favors pellets formation of about 1 mm in diameter. Besides, the specific citric acid production (g citric acid per g biomass dry mass) increases by 40%, while the volumetric productivity (g citric acid per l per h) and the yield (g citric acid per g initial sucrose) are doubled (Pera and Callieri 1997). On the other hand, the dispersed mycelium obtained in the presence of 1 g l^-1^ FeCl_3_ is preferred for the production of both a bioemulsifier (Colin et al. 2010a) and a mycelium-bound transesterification lipase activity (Colin et al. 2011).

Concerning microscopic morphology, this work explored a new experimental procedure in order to quantitatively check the potential of some culture conditions to induce a determined hyphal morphology by using a Mb-NAGase activity as a biomarker. Interestingly, a highly negative correlation between the l_HGU_ and the specific Mb-NAGase activity was found. In fact, the environment was able to induce highly branched mycelia only under those culture conditions compatible with specific Mb-NAGase values equal to or higher than 190 U g _dry_._wt_^-1^. These results could also indicate that the fungus adjusts its morphology in response not only to the surrounding substrate concentration (Lübbehüsen et al. 2004; Ziv et al. 2008), but also to the environmental culture condition as a whole.

In connection to the involving of wall lytic enzymes in hyphal morphology, a decrease of the l_HGU_ is reported in some chitin synthase mutants as well. The l_HGU_ for a ChsB/G (*chsB* disruption) *Aspergillus oryzae* strain is 52% lower than that of the wild type (A1560). In addition, the hyphal diameter in that mutant is significantly higher than in the A1560 strain (Müller et al. 2002). A similar morphological response is caused by the repression of a *chsB* gene expression. Ichinomiya et al. (2002) constructed a *chsB*-conditional mutant in which *chsB* is placed under an inducible promoter, such as the alcohol dehydrogenase gene promoter of *Aspergillus nidulans*. Thus, under repressing conditions, the mutant produces highly branched hyphae. These results could be also compatible with an increase of the wall lytic potential.

Results previously reported (Pera et al. 1997) as well as the experimentation presented here constitute evidence to support the following simple quantitative approach to tailor a desired fungal microscopic growth. It basically consists of three major steps: 1) to select a relative marker of the wall lytic potential, 2) to conduct *in vitro* dose–response assays of potential modifiers of the selected enzyme marker, and 3) to design fermentation conditions using those effectors that are compatible with the desired microscopic morphology. This methodology was successfully applied in some biotechnological processes involving eukaryotic cells. One of them is the improvement of citric acid production using *A. niger* MYA 135. It has long been established that highly branched mycelium and bulbous cells are associated with good citric acid yields (Papagianni 2007). In this connection, the presence of CaCl_2_ not only increases a NAGase purified extract activity (Pera et al. 1997) but also the addition of 0.5 g l^-1^ of this salt to the fermentation medium induces a highly branched mycelium with abundant bulbous cells. In addition, under this culture condition the uptake of phosphate and sucrose as well as the production of citric acid (90 g l^-1^) are increased by 15, 35 and 50%, respectively (Pera and Callieri 1997). On the contrary, the presence of FeCl_3_ in a range of 0.5 - 10.0 mM impaired a NAGase purified extract activity (Pera et al. 1997). Thus, as it was mentioned before, the addition of 1 g l^-1^ FeCl_3_ to the BM encourages a scarcely branched mycelium and increases the production of both a mycelium-bound transesterification lipase activity (Colin et al. 2011) and an emulsifier compound (Colin et al. 2010a). Interestingly, in the same medium supplemented with 2% olive oil it was also observed that a specific extracellular hydrolytic lipase activity was increased by 6.6 fold after four days of incubation compared to the control (Colin et al. 2010b). Finally, another application is the enhancement of protoplast formation in *Phaffia rhodozyma*. This yeast displays an *in situ* β-D-glucosidase activity that is clearly increased by the presence of MnCl_2_. When *P. rhodozyma* grows in the presence of 4.0 mM MnCl_2_, the cells are bigger and rounder than those obtained in its absence, suggesting a weakening of the cell wall. Protoplasts are produced from these cells using KCl as an osmotic stabilizer (Pera et al. 1999). It might also be mentioned that *P. rhodozyma* feeding cells with weakened walls increases the efficiency of deposition of astaxanthin in the flesh of cultivated salmonids and crustaceans (Johnson 1989).

On the other hand, rheology-hyphal micromorphology relationships are particularly relevant in fermentation involving filamentous fungi. Bocking et al. (1999) reported that cultures of highly branched mutants are less viscous than those of the parental strains. They also found a linear relationship between the l_HGU_ and the broth viscosity. In addition, Metz et al. (1981) in studies with *Penicillium chrysogenum* observed that the length of the hyphal elements decreased with increasing the power input per unit mass, as the increased agitation caused the hyphae to become shorter, thicker and highly branched. However, van Suijdan and Metz (1981) also found that the energy input required to reduce the hyphal length sufficiently to achieve a significant reduction in broth viscosity is enormous, and so broth viscosity reduction by this technique seems of little practical value. In this connection, the induction of a desired fungal microscopic pattern by permissible modification of environmental conditions could overcome that observation.

In summary, this work showed that both conidia adhesion capacity and hyphal morphology were among the governing factors affecting the final pellet diameter. It was also observed that once the pellet was formed the l_HGU_ had an important influence on its final diameter. In addition, it was explored a new experimental procedure in order to quantitatively check the potential of some culture conditions to induce a determined hyphal morphology by using a Mb-NAGase activity as a biomarker. Besides, a practical and a quantitative method to check in few minutes the conidia adhesion capacity was also proposed. On the other hand, the information obtained was used to design culture conditions compatible with a desired form of fungal growth. Appropriate fungal morphologies are already successfully applied in some biotechnological processes.

Finally, if the results reported in the present article prove to be applicable to other fungal strains, manipulation of fungal morphology will be easier in industrial filamentous fungal fermentation.

## Competing interest

The authors declare that they have no competing interest.

## References

[B1] Amicucci A Balestrini R Kohler A Barbieri E Saltarelli R Faccio A Roberson RW Bonfante P Stocchi V Hyphal and citoskeleton polarization in *Tuber melanosporum*: A genomic and cellular analysis Fungal Genet Biol 2011 3 561 572 10.1016/j.fgb.2010.12.002 21176788

[B2] Amiri A Cholodowski D Bompeix G Adhesion and germination of waterborne and airborne conidia of *Penicillium expansum* to apple and inert surfaces Physiol Mol Plant Pathol 2005 3 40 48 10.1016/j.pmpp.2005.07.003

[B3] Bartnicki-García S Lippman E The bursting tendency of hyphal tips of fungi: presumptive evidence for a delicate balance between wall synthesis and wall lysis in apical growth J Gen Microbiol 1972 3 487 500

[B4] Blánquez P Caminal G Sarrà M Vincent T The effect of HRT on the decolourisation of the Grey Lanaset G textile dye by *Trametes versicolor* Chem Eng J 2007 3 163 169 10.1016/j.cej.2006.09.007

[B5] Bocking SP Wiebe MG Robson GD Hansen K Christiansen LH Trinci APJ Effect of branch frequency in *Aspergillus oryzae* on protein secretion and culture viscosity Biotechnol Bioeng 1999 3 638 648 10.1002/(SICI)1097-0290(19991220)65:6<638::AID-BIT4>3.0.CO;2-K 10550770

[B6] Colin VL Baigorí MD Pera LM Bioemulsifier production by *Aspergillus niger* MYA 135: presumptive role of iron and phosphate on emulsifying ability World J Microbiol Biotechnol 2010 3 2291 2295 10.1007/s11274-010-0409-4

[B7] Colin VL Baigorí MD Pera LM Effect of environmental conditions on extracellular lipases production and fungal morphology from *Aspergillus niger* MYA 135 J Basic Microbiol 2010 3 52 58 10.1002/jobm.200900162 20082373

[B8] Colin VL Baigorí MD Pera LM Mycelium-bound lipase production from *Aspergillus niger* MYA 135, and its potential applications for the transesterification of ethanol J Basic Microbiol 2011 3 236 242 10.1002/jobm.201000232 21298682

[B9] Doss RP Potter SW Chastagner GA Christian JK Adhesion of nongerminated *Botrytis cinerea* conidia to several substrata Appl Environ Microbiol 1993 3 1786 1791 16348954 10.1128/aem.59.6.1786-1791.1993 PMC182162

[B10] Dynesen J Nielsen J Surface hydrophobicity of *Aspergillus nidulans* conidiospores and its role in pellets formation Biotechnol Prog 2003 3 1049 1052 10.1021/bp0340032 12790678

[B11] El-Enshasy H Kleine J Rinas U Agitation effects on morphology and protein productive fractions of filamentous and pelleted growth forms of recombinant *Aspergillus niger* Process Biochem 2006 3 2103 2112 10.1016/j.procbio.2006.05.024

[B12] Fontaine T Beauvais A Loussert C Thevenard B Fulgsang CC Ohno N Clavaud C Prevost M Latgé J Cell wall α 1–3 glucans induce the aggregation of germinating conidia of *Aspergillus fumigatus* Fungal Genet Biol 2010 3 707 712 10.1016/j.fgb.2010.04.006 20447463

[B13] Grimm LH Kelly S Krull R Hempel DC Morphology and productivity of filamentous fungi Appl Microbiol Biotechnol 2005 3 375 384 10.1007/s00253-005-0213-5 16317480

[B14] Grove SN Smith JE, Berry DR The cytology of hyphal tip growth The filamentous fungi 1978 Arnold, London

[B15] Ichinomiya M Motoyama T Fujiwara M Takagi M Horiuchi H Ohta A Repression of *chsB* expression reveals the functional importance of class IV chitin synthase gene *chsD* in hyphal growth and conidiation of *Aspergillus nidulans* Microbiology 2002 3 1335 1347 11988507 10.1099/00221287-148-5-1335

[B16] Johnson EA Yeast: A pigment source in salmonids Feed Management 1989 3 18 21

[B17] Krull R Wucherpfenning T Esfandabadi ME Walisko R Melzer G Hempel DC Kampen I Kwade A Wittmann C Characterization and control of fungal morphology for improved production performance in biotechnology J Biotechnol 2013 3 112 123 10.1016/j.jbiotec.2012.06.024 22771505

[B18] Lübbehüsen T González Polo V Rossi S Nielsen J Moreno S McIntyre M Arnau J Protein kinase A is involved in the control of morphology and branching during aerobic growth of *Mucor circinelloides* Microbiology 2004 3 143 150 10.1099/mic.0.26708-0 14702407

[B19] Mercure EW Leite B Nicholson RL Adhesion of ungerminated conidia of Colletotrichum graminicola to artificial hydrophobic surfaces Physiol Mol Plant Pathol 1994 3 421 440 10.1016/S0885-5765(05)80040-2

[B20] Metz B de Bruijn EW van Suijdam JC Method for quantitative representation of the morphology of molds Biotechnol Bioeng 1981 3 149 162 10.1002/bit.260230110

[B21] Meyer V Genetic engineering of filamentous fungi-Progress, obstacles and future trends Biotechnol Adv 2008 3 177 185 10.1016/j.biotechadv.2007.12.001 18201856

[B22] Mizunuma T Kokufuta E Sato S A mycelium with polyelectrolyte complex-bunched hyphae: Preparation and fermentation performance Colloids Surf B 2007 3 155 160 10.1016/j.colsurfb.2006.11.011 17182227

[B23] Müller C McIntyre M Hansen K Nielsen J Metabolic engineering of the morphology of *Aspergillus oryzae* by altering chitin synthesis Appl Environ Microbiol 2002 3 1827 1836 10.1128/AEM.68.4.1827-1836.2002 11916702 PMC123896

[B24] Newey LJ Caten CE Green JR Rapid adhesion of *Stagonospora nodorum* spores to a hydrophobic surface requires pre-formed cell surface glycoproteins Mycol Res 2007 3 1255 1267 10.1016/j.mycres.2007.09.007 17998157

[B25] Papagianni M Fungal morphology and metabolite production in submerged mycelial processes Biotechnol Adv 2004 3 189 259 10.1016/j.biotechadv.2003.09.005 14665401

[B26] Papagianni M Advances in citric acid fermentation by *Aspergillus niger*: biochemical aspects, membrane transport and modeling Biotechnol Adv 2007 3 244 263 17337335 10.1016/j.biotechadv.2007.01.002

[B27] Papagianni M Mattey M Physiological aspects of free and immobilized *Aspergillus niger* cultures producing citric acid under various glucose concentration Process Biochem 2004 3 1963 1970 10.1016/j.procbio.2003.09.027

[B28] Pera LM Callieri DAS Influence of calcium on fungal growth, hyphal morphology and citric acid production in *Aspergillus niger* Folia Microbiol 1997 3 551 556 10.1007/BF02815463 9438355

[B29] Pera LM Infante Majolli MV Baigorí MD Purification and characterization of a thermostable and highly specific β-N-Acetyl-D-glucosaminidase from *Aspergillus niger* 419 Biotechnol Appl Biochem 1997 3 183 187 9428156

[B30] Pera LM Rubinstein L de Figueroa LIC Baigorí MD Callieri DAS Influence of manganese on cell morphology, protoplast formation and ß-D-glucosidase activity in *Phaffia rhodozima* FEMS Microbiol Lett 1999 3 155 160

[B31] Pera LM Baigorí MD Castro GR Pandey A, Larroche C, Soccol CR Biotransformations Advances in Fermentation Technology 2008 Asiatech Publishers, New Delhi

[B32] Pera LM Luna F Castro GR Baigorí MD Krause J, Fleischer O Tailoring of industrial products by submerged fermentation Industrial Fermentation: Food Processes, Nutrient Sources and Production Strategies 2010 Nova Science Publishers, New York

[B33] Prosser JI Tough AJ Growth mechanisms and growth kinectis of filamentous Microorganisms Crit Rev Biotechnol 1991 3 253 273 10.3109/07388559109038211 2070422

[B34] Rittenour WR Si H Harris SD Hyphal morphogenesis in *Aspergillus nidulans* Fungal Biol Rev 2009 3 20 29 10.1016/j.fbr.2009.08.001

[B35] Romero S Blánquez P Caminal G Font X Sarrà M Gabarrell X Vincent T Different approaches to improving the textile dye degradation capacity of *Trametes versicolor* Biochem En J 2006 3 42 47 10.1016/j.bej.2006.05.018

[B36] Schumacher CFA Steiner U Dehne H Oerke E Localized adhesion of nongerminated *Venturia inaequalis* conidia to leaves and artificial surfaces Phytopathology 2008 3 760 768 10.1094/PHYTO-98-7-0760 18943251

[B37] Shoji J Arioka M Kitamoto K Vacuolar membrane dynamics in the filamentous fungus *Aspergillus oryzae* Eukaryot Cell 2006 3 411 421 10.1128/EC.5.2.411-421.2006 16467481 PMC1405889

[B38] Slawecki RA Ryan EP Young DH Novel fungitoxicity assays for inhibition of germination-associated adhesion of *Botrytis cinerea* and *Puccinia recondite* spores Appl Environ Microbiol 2002 3 597 601 10.1128/AEM.68.2.597-601.2002 11823196 PMC126693

[B39] Smith SN Chohan R Armstrong RA Whipps JM Hydrophobicity and surface electrostatic charge of conidia of the mycoparasite *Coniothyrium minitans* Mycol Res 1998 3 243 249 10.1017/S0953756297004796

[B40] Van Suijdam JC Metz B Influence of engineering variables upon the morphology of filamentous molds Biotechnol Bioeng 1981 3 111 148 10.1002/bit.260230109

[B41] Wucherpfennig T Hestler T Krull R Morphology engineering – osmolality and its effect on *Aspergillus niger* morphology and productivity Microb Cell Fact 2011 3 58 10.1186/1475-2859-10-58 21801352 PMC3178489

[B42] Zelinger E Hawes CR Gurr SJ Dewey FM Attachment and adhesion of conidia of *Stagonospora nodorum* to natural and artificial surfaces Physiol Mol Plant Pathol 2006 3 209 215 10.1016/j.pmpp.2006.11.002

[B43] Ziv C Gorovits R Yarden O Carbon source affects PKA-dependent polarity of *Neurospora crassa* in a CRE-1-dependent and independent manner Fungal Genet Biol 2008 3 103 116 10.1016/j.fgb.2007.05.005 17625933

[B44] Žnidaršič P Pavko A The morphology of filamentous fungi in submerged cultivations as a bioprocess parameter Food Technol Biotechnol 2001 3 237 252

